# Transcranial direct current stimulation for promoting motor function in cerebral palsy: a review

**DOI:** 10.1186/s12984-018-0476-6

**Published:** 2018-12-20

**Authors:** Melanie K. Fleming, Tim Theologis, Rachel Buckingham, Heidi Johansen-Berg

**Affiliations:** 1 Wellcome Centre for Integrative Neuroimaging, FMRIB, Nuffield Department of Clinical Neurosciences, University of Oxford, John Radcliffe Hospital, Oxford, OX3 9DU UK; 2 Nuffield Orthopaedic Centre, Oxford University Hospitals NHS Foundation Trust, Oxford, UK

**Keywords:** Cerebral palsy, Motor function, Transcranial direct current stimulation, Brain stimulation, Upper limb, Lower limb

## Abstract

Transcranial direct current stimulation (tDCS) has the potential to improve motor function in a range of neurological conditions, including Cerebral Palsy (CP). Although there have been many studies assessing tDCS in adult stroke, the literature regarding the efficacy of tDCS in CP is more limited. This review therefore focuses on the neurophysiological and clinical findings in children and adolescents with CP. Initial studies applying anodal tDCS to promote lower limb function are promising, with improvements in gait, mobility and balance reported. However, the results of upper limb studies are mixed and more research is needed. Studies investigating neurophysiological changes or predictors of response are also lacking. Large-scale longitudinal studies are needed for the lower limb to ascertain whether the initial pilot results translate into clinically meaningful improvements. Future studies of the upper limb should focus on determining the optimal stimulation parameters and consider tailoring stimulation to the individual based on the (re)organisation of their motor system.

## Introduction

**Fig. 1:**
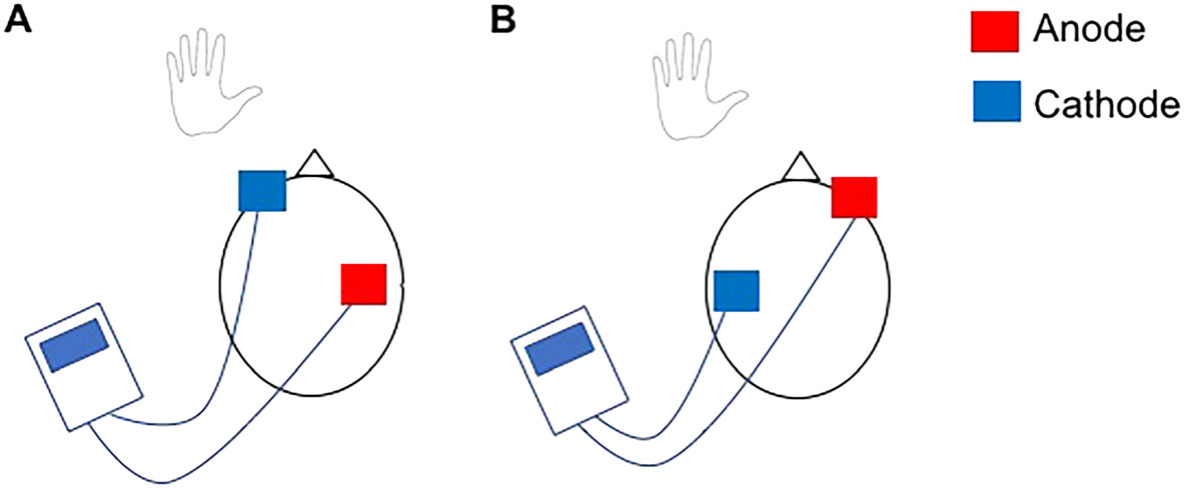
Diagrammatic representation of tDCS. **a** Anodal stimulation applied over the motor cortex contralateral to the trained limb. **b** Cathodal stimulation applied over the motor cortex ipsilateral to the trained limb, based on the interhemispheric imbalance model

Transcranial direct current stimulation (tDCS), a form of non-invasive brain stimulation, has received considerable interest as a neuromodulatory technique with the potential to enhance cortical plasticity and improve motor function in a range of neurological conditions. Low intensity, direct, constant current is applied to the scalp (Fig. 1), typically over the primary motor cortex (M1), and cortical excitability and inhibition is altered depending on the stimulation parameters [1, 2] (Table 1).

**Table 1:** Fundamentals of tDCS

• Typically, two electrodes are placed on the scalp, one over the area of interest (e.g. motor cortex), and current flows between them • Typical sensations include tingling, prickling and itching of the scalp as the current intensity ramps up • Sham stimulation can be effectively applied by ramping up stimulation for a short period, then turning it off • Current direction, duration and intensity all require consideration • Anodal tDCS typically enhances cortical excitability and reduces inhibition, cathodal tDCS typically suppresses excitability • tDCS can be delivered at rest or during a task, e.g. motor training • Effects can be seen during and after tDCS • Single session effects can be seen, or cumulative effects over multiple sessions	

One of the neurological conditions which may benefit from the neuromodulatory effects of tDCS is Cerebral Palsy (CP), whereby motor function and development are affected by an insult to the developing brain [3]. Since functional deficits limit independence and quality of life long term, the potential to utilise tDCS as an adjunct to physical therapy for enhancing motor function is an attractive concept. However, although there has been considerable investigation of the effectiveness of tDCS for adult stroke, the findings cannot be assumed to translate directly into children and adolescents with CP, due to differences in brain size, maturity, anatomy and reorganisation. The application of tDCS in this population appears to be safe [4] and safety guidelines have been developed [5]. This narrative review therefore focuses on the neurophysiological and clinical findings with use of tDCS in children and adolescents (6–21 years) with CP.

## Information sources

References for this review were identified, by MF, through searches of PubMed for articles published up to July 2018. Combinations of the terms “cerebral palsy”, “tdcs”, “brain stimulation”, “child stroke” and “pediatric stroke” were used. Additionally, articles were identified through article reference lists. The final reference list was selected, by MF, on the basis of topic relevance.

## Neurophysiological findings

Assessment of change in cortical activity or excitability is important in order to understand the mechanism of action of tDCS. Additionally, differences in neurophysiological outcomes may potentially be of use to explain variability in clinical outcome, while variations in neurophysiological measures at baseline may be able to predict who will benefit from tDCS. Currently, there are very few studies in CP which have reported using brain imaging or neurophysiological measures alongside tDCS.

TDCS is known to alter cortical excitability, intracortical inhibition, and cortical plasticity [1, 2, 6, 7] and these neuromodulatory effects are thought to underlie the behavioural or clinical efficacy of tDCS. Transcranial Magnetic Stimulation (TMS) is commonly used to assess changes in cortical excitability or intracortical inhibition following a single session of tDCS in adult stroke [8, 9]. However, to our knowledge, there are no published studies of this type in CP. One study [10] reported an increase in motor evoked potential (MEP) amplitude elicited by TMS following 10 days of anodal tDCS (1 mA, 20 min) targeting the lower limb. MEPs were elicited at 110% rest motor threshold (RMT) from the abductor muscle of the thumb and the quadriceps muscle of the lower limb at rest. Each hemisphere was stimulated separately, but the results do not separate the findings from each muscle or hemisphere. Therefore, although anodal tDCS appeared to increase cortical excitability, as hypothesised, it is unclear as to how specific the changes are to the targeted region or the time-scale over which these changes occurred.

Changes in brain metabolites following tDCS can be assessed using Magnetic Resonance Spectroscopy (MRS) [2, 11, 12]. This can provide insights into alterations in measures of neuronal health or changes in levels of cortical inhibitory or excitatory neurotransmitters. Auvichayapat et al. [13] attempted to assess changes in brain metabolites following tDCS using MRS in children with CP. Anodal tDCS (20 min, 1 mA) was delivered for 5 consecutive days to the left M1 in children 8–12 years old with spastic CP affecting their right upper limb. They reported a significant increase in concentrations of N-acetylaspartate (NAA), Choline and Myoinositol in the left basal ganglia and an increase in the ratio of Glx (a combination of glutamate and glutamine) to Creatine in the left M1. Although there was no sham control group, the authors speculated that the tDCS-induced increase in activity of the M1 leads to an increase in the concentration of NAA, Choline and Myoinositol in the basal ganglia. There was a negative correlation between the ratio of Glx:Creatine in the M1 and the spasticity (Tardieu scale score) of the right upper limb (shoulder flexors, shoulder external rotators, elbow flexors and elbow pronators) following tDCS. However, the authors did not report whether this relationship existed at baseline or whether the change in metabolite ratios correlated with change in spasticity. There was also no indication of the quality of the MRS data, which is typically an important consideration in MRS studies. High quality MRS data may be difficult to obtain in this population, especially in regions such as the basal ganglia.

## Upper limb function

Research on the effect of tDCS on upper limb function in CP is limited to date (Table 2). Similar to adult stroke [14] the studies that have been published have utilised the “interhemispheric imbalance model” as rationale. The interhemispheric imbalance model proposes that there are abnormal levels of interhemispheric inhibition from the contralesional to ipsilesional M1, resulting in a reduction in activity of the ipsilesional M1 during movement of the affected limb and an increase in activity of the contralesional M1 [15–17]. Therefore, this model provides rationale for applying anodal tDCS to the ipsilesional M1 to increase excitability, or cathodal tDCS to the contralesional M1 in an attempt to decrease excitability and thereby upregulate the ispilesional M1 through a reduction in interhemispheric inhibition from the contralesional hemisphere.

**Table 2:** Summary of study methods and findings for studies targeting the upper limb

	Electrode montage	Intensity	Electrode area	Control	No. sessions	Stimulation duration	Participants	N	Motor training	Summary of findings
Moura et al., 2017 [18]	Anode ipsilesional M1, Cathode contralateral supraorbital ridge	1 mA	25 cm^2^	Sham group	1	20 min	Spastic hemiplegia, 6–12 years	10 per group	20 min reaching training with constraint	↓ total movement duration during reaching. No other changes
Auvichayapat et al., 2017 [13]	Anode left M1, Cathode contralateral shoulder	1 mA	35 cm^2^	None	5	20 min	Hemiparetic/ diparetic with spasticity of right UL, 8–12 years	10	Not mentioned	↓ Tardieu score ↑ one aspect of QUEST
Aree-Uea et al., 2014 [19]	Anode left M1, Cathode right shoulder	1 mA	35 cm^2^	Sham group	5	20 min	Spastic hemiplegia of right UL, 8–18 years	23 per group	Physical therapy, including stretches	↓ MAS ↑ passive shoulder abduction ROM
Gillick et al., 2018 [20]	Cathode contralesional M1, Anode contralateral supraorbital ridge	0.7 mA	Not specified	Sham group	10	20 min	Hemiparetic, 7–21 years	10 per group	120 min CIMT	↑ AHA, no difference between groups. No change COPM
Kirton et al., 2017 [21]	Cathode contralesional, Anode contralateral supraorbital ridge	1 mA	35 cm^2^	Sham group	10	20 min	Hemiparetic, 6–18 years	12 active, 11 sham	120 min CIMT	↑ COPM

*UL* Upper limb, *CIMT* Constraint induced movement therapy, *QUEST* Quality of upper extremity skills test, *MAS* Modified Ashworth scale, *ROM* Range of movement, *AHA* Assisting hands assessment, *COPM* Canadian occupational performance measure

A single session study [18] delivered 20 min of 1 mA anodal tDCS (or sham) to the ipsilesional M1 of children with spastic hemiplegia, alongside 20 min of motor training of the affected arm with constraint of the other arm. Using motion analysis, a significant reduction in total movement duration during reaching movements with the affected hand was observed for the tDCS group compared with sham. Although this initially seems promising, there were numerous comparisons made, and none of the other change values (e.g. smoothness, velocity or accuracy parameters) showed significant between-group differences.

Two studies have delivered multiple sessions of anodal tDCS in CP [13, 19]. Auvichayapat et al. [13] delivered 5 days of tDCS to the left M1. Although there was a mixture of hemiparetic and diparetic participants, all had spasticity of their right upper limb. However, there is no mention as to whether there was any motor training alongside the tDCS. Although the authors reported an improvement in spasticity (Tardieu scale) and one aspect of the Quality of Upper Extremity Skills Test (QUEST), there was no sham group for comparison. A randomised, double-blinded study [19] aimed to assess changes in spasticity with 5 consecutive days of anodal tDCS (20 min, 1 mA) to the left M1 of children with spastic hemiplegia affecting the right arm. In addition to the tDCS, participants engaged in “routine physical therapy”, including passive and active stretching, therapeutic positioning and aerobic exercise. There were improvements in spasticity of the shoulder, elbow, wrist and fingers and an improvement in shoulder abduction passive range of movement for the active tDCS group only. However, there were no active motion function measures assessed.

Two double-blind randomised trials [20, 21] have combined 20 min of cathodal tDCS of the contralesional M1 with motor training, including constraint induced movement therapy (CIMT), over 10 sessions in children with hemiparetic CP. Both active and sham groups demonstrated a significant increase in the Assisting Hand Assessment (AHA), which measures bimanual function during novel play or functional tasks, but there was no difference between groups. Kirton et al. [21] did find greater improvement in self-reported performance (using the Canadian Occupational Performance Measure (COPM)) for the active tDCS group, and a higher proportion of participants achieved a clinically significant improvement on this measure compared to the sham group. However, the COPM did not show between-group differences in the study by Gillick et al. [20], indicating that more research is needed with both objective and subjective measures.

The intensity of the current for cathodal tDCS may be an issue in the studies so far. Contrary to effects in adults [1], in a study with healthy children (11–16 years) [22], corticospinal excitability was found to increase, rather than decrease, following 1 mA cathodal tDCS. If the intensity of stimulation was lowered to 0.5 mA then the hypothesised decrease in MEP amplitude for cathodal tDCS was evident. Moliadze et al. therefore speculated that 0.5 mA cathodal stimulation in children may produce similar effects as 1 mA in adults. The situation is different from anodal stimulation: 0.5 mA anodal stimulation was found to be ineffective at increasing MEP amplitude in children whereas 1 mA anodal stimulation did lead to a significant increase [22], consistent with effects of anodal tDCS in adults [1].

Therefore, there is currently no indication that tDCS provides additional benefit for active motor function over motor training or CIMT alone in children and young people with CP, but spasticity appears to improve with anodal tDCS.

## Lower limb function

A summary of studies delivering tDCS to target lower limb function is provided in Table 3. To our knowledge, there are only two single-session studies of tDCS in CP targeting lower limb function [23, 24]. One study [23] delivered 20 min of anodal tDCS (1 mA) at rest to the dominant hemisphere in participants with hemiparetic or diparetic CP. Motion analysis was used to assess balance and gait before, immediately following and 20 min later. There was a significant reduction in sway and an increase in walking speed for the active group compared with sham, but no change in cadence. However, the results of the study by Lazzari et al. [24] are less promising. Anodal tDCS was delivered to the motor cortex (the authors do not specify which hemisphere) for 20 min (1 mA) in combination with 20 min of mobility training using virtual reality (Xbox 360 with Kinect movement sensor (Microsoft Corporation, Redmond, WA)). The virtual reality training involved walking with and without simulated obstacles. Static balance was assessed using a force plate. The authors report an increase in sway velocity immediately following the intervention for both groups, but no clear between-group differences. There was no later follow-up assessment and therefore the increase in sway velocity observed immediately could represent a deterioration in balance due to fatigue for both groups following the mobility training. If this is the case, then it would appear that tDCS was not effective at ameliorating this fatigue effect.

**Table 3:** Summary of study methods and findings for studies targeting the lower limb

	Electrode montage	Intensity	Electrode area	Control	No. sessions	Stimulation duration	Participants	N	Motor training	Summary of findings
Grecco et al., 2014 [23]	Anode dominant M1, Cathode contralateral supraorbital ridge	1 mA	25 cm^2^	Sham group	1	20 min	Hemiparetic/ diparetic, 4–12 years	10 per group	At rest	↓ sway ↑ walking speed No change cadence
Lazzari et al., 2015 [24]	Anode M1 (laterality not specified), Cathode contralateral supraorbital ridge	1 mA	25 cm^2^	Sham group	1	20 min	4–12 years (other details not specified)	10 per group	20 min mobility training using VR	↑ sway velocity both groups
Collange Grecco et al., 2015 [10]	Anode M1 contralateral to lower limb with most impairment, Cathode contralateral supraorbital ridge	1 mA	25 cm^2^	Sham group	10	20 min	Spastic diparetic, 5–10 years	10 per group	20 min VR gait training	↑ walking velocity and cadence ↑ PEDI (mobility)
Duarte et al., 2014 [25]	Anode M1 ipsilateral to dominant limb, or ipsilesional, Cathode contralateral supraorbital ridge	1 mA	25 cm^2^	Sham group	10	20 min	Spastic hemiparetic/ diparetic, 5–10 years	12 per group	20 min Treadmill training	↑ PBS lower sway for active vs sham post-intervention ↑ PEDI for active group, but no between-group differences
Lazzari et al., 2017 [26]	Anode M1 (laterality not specified), Cathode contralateral supraorbital ridge	1 mA	25 cm^2^	Sham group	10	20 min	4–12 years (other details not specified)	10 per group	20 min VR mobility training	↑ PBS and TUG ↓ oscillation of centre of pressure

*VR* Virtual reality, *PBS* Pediatric balance scale, *PEDI* Pediatric evaluation disability inventory, *TUG* Timed up and go

Three studies have assessed multiple sessions of anodal tDCS for promoting lower limb function [10, 25, 26]. Duarte et al. [25] delivered 10 sessions of anodal tDCS (1 mA, 20 min), in combination with treadmill training in children with spastic CP. There was a mixture of hemiparetic and diparetic participants and the anode was placed over the motor cortex ipsilateral to the dominant limb (thereby stimulating the more-affected hemisphere). Interpretation is complicated as the authors report within group changes and between group score comparisons separately, rather than using a mixed analysis of variance or change scores. Nonetheless, within-group comparisons demonstrated an improvement in the Pediatric Balance Scale (PBS) for the active tDCS group only, and between-group comparisons showed that the active group had a higher PBS score and lower sway than the sham group when assessed following the intervention and at the 1 month follow-up. Similarly, there was an improvement for the active group on the mobility subsection of the Pediatric Evaluation Disability Inventory (PEDI), which is a subjective assessment of functional performance in activities of daily living. However, the scores did not differ between groups.

Collange Grecco et al. [10] used virtual reality for gait training in combination with 10 sessions of anodal tDCS (1 mA, 20 min) in children with spastic diparetic CP. The virtual reality training involved walking around a simulated race track at varying speeds (Xbox 360 with Kinect movement sensor (Microsoft Corporation, Redmond, WA)). Participants were asked which lower limb they found had most difficulty during gait and the anode was placed over the contralateral motor cortex. Their primary outcome measure was gait kinematics, using motion analysis. There was a greater improvement in walking velocity and cadence for the tDCS group compared to sham, but not for any of the other gait variables assessed. Mobility, assessed using the PEDI, also improved for the active tDCS group, but not for sham. Similarly promising results were found by Lazzari et al. [26], who combined anodal tDCS (1 mA, 20 min) with 20 min of mobility training using virtual reality over 10 sessions. The virtual reality training involved a game that simulates stationary walking requiring complete flexion of the hip, knee and ankle, and weight transfer from one limb to the other (Xbox 360 with Kinect movement sensor (Microsoft Corporation, Redmond, WA)). They demonstrated a significantly greater improvement in the PBS and the Timed Up and Go (TUG) for the active tDCS group compared with sham. There was also a greater improvement in static balance, assessed as the oscillation of the centre of pressure. However, variability within groups was high and there was no investigation of variables to account for variability.

## Predictors of response

Data on the predictors of response to tDCS are currently lacking in this population. The only study to attempt to analyse potential predictors [27] did so by combining 3 studies that delivered anodal tDCS alongside gait training (for a total of 56 participants) in children with spastic hemiparetic or diparetic CP. The authors reported that two predictors were significantly associated with the responsiveness to the intervention; MEP presence during initial evaluation (indicating preservation of the corticospinal tract) and location of the injury (cortical or subcortical). However, it is unclear whether this is specific to the modulatory effects of the tDCS *per se* or simply an indicator of who has the potential to improve motor function, as is the case for adult stroke survivors [28].

For the upper limb, it is currently unclear whether anodal or cathodal tDCS should be applied in unilateral CP. Indeed, this decision may depend on the extent to which the activity of each M1 is required for motor function, based on the degree to which the ipsilesional M1 and corticospinal tract are damaged. Although in some instances, over-activity of the contralesional hemisphere may be maladaptive [29] and benefit from downregulation, in other cases the motor system may be reorganised towards ipsilateral control [30, 31]. If the control of the paretic hand is through fast-conducting ipsilateral projections from the contralesional M1, then attempting to enhance ipsilesional M1 excitability with anodal tDCS may be futile. Equally, decreasing excitability of the contralesional M1 with cathodal tDCS might be detrimental, as is seen for people with severe upper limb impairment after adult stroke [32]. It is difficult to determine whether someone with CP relies on ipsilateral control from the contralesional hemisphere based on clinical presentation alone, as children with ipsilateral projections can show a useful grasp, or no movement at all [30]. Therefore, measures, such as Diffusion Tensor Imaging (DTI) to assess fractional anisotropy of the corticospinal tract, or TMS to assess corticospinal tract integrity through the presence or absence of MEPs, may be necessary for informing choices with regard to electrode placement. We therefore propose that future studies attempt to optimise tDCS delivery, based on knowledge of the (re)organisation of the individual’s motor system.

There is currently no investigation into the optimal age for delivery of tDCS. The studies presented here have delivered tDCS to children and adolescents, but it is conceivable that the responsiveness could be dependent on the stage of development of the individual. Therefore, future studies are needed to address this issue.

## Conclusions

Application of tDCS for enhancing lower limb function in young people with CP appears effective, although large-scale longitudinal studies are required to confirm the initially promising findings. Further single-session and longitudinal studies are required to determine the efficacy of tDCS for the upper limb and to elucidate mechanisms of action and predictors of response in this population.

## Abbreviations

AHA: Assisting Hand Assessment
CIMT: Constraint induced movement therapy
COPM: Canadian Occupational Performance Measure
CP: Cerebral Palsy
DTI: Diffusion Tensor Imaging
M1: Primary motor cortex
MEP: Motor evoked potential
MRS: Magnetic Resonance Spectroscopy
NAA: N-acetylaspartate
PBS: Pediatric Balance Scale
PEDI: Pediatric Evaluation Disability Inventory
QUEST: Quality of Upper Extremity Skills Test
RMT: Rest motor threshold
tDCS: Transcranial direct current stimulation
TMS: Transcranial magnetic stimulation
TUG: Timed Up and Go
